# Metal Coated Polypropylene Separator with Enhanced Surface Wettability for High Capacity Lithium Metal Batteries

**DOI:** 10.1038/s41598-019-53257-4

**Published:** 2019-11-14

**Authors:** Mir Mehraj Ud Din, Ramaswamy Murugan

**Affiliations:** High Energy Density Batteries Research Laboratory, Department of Physics, Pondicherry University, Puducherry, 605014 India

**Keywords:** Energy science and technology, Batteries

## Abstract

Lithium metal batteries are among the strong contenders to meet the increasing energy demands of the modern world. Metallic lithium (Li) is light in weight, possesses very low standard negative electrochemical potential and offers an enhanced theoretical capacity (3860 mA h g^−1^). As a negative electrode Li paves way to explore variety of elements including oxygen, sulfur and various other complex oxides as potential positive electrodes with a promise of much higher energy densities than that of conventional positive electrodes. However, there are technical challenges in utilizing metallic lithium due to its higher reactivity towards liquid electrolytes and higher affinity to form Li dendrites, leading to serious safety concerns. Here, we report on preparation of niobium (Nb) metal-coated binder-free and highly hydrophilic polypropylene separator prepared via radio frequency (RF) magnetron sputtering. Thin layer of niobium metal (Nb) particles were deposited onto the polypropylene (PP) sheet for various time periods to achieve desired coating thickness. The as-prepared separator revealed excellent hydrophilic behaviour due to enhanced surface wettability. Symmetric cells display reduced interface resistance and uniform voltage profiles for 1000 cycles with reduced polarization at higher current densities suggesting smooth stripping and plating of Li and homogeneous current distribution at electrode/electrolyte interface under room temperature conditions. Nb nanolayer protected separator with LiNi_0.33_M_0.33_Co_0.33_O_2_ (LNMC) and composite sulfur cathodes revealed an enhanced cycling stability.

## Introduction

Current research in lithium ion batteries (LiBs) is mainly focused on utilization of metallic lithium (Li) as anode material for developing advanced high energy density lithium metal batteries based on conventional insertion-type cathodes and several other high capacity cathode materials. LiCoO_2_ is widely used as cathode material in LiBs due to its reasonable cycling stability^1–6^. However, higher cost, toxicity and lower practical capacity limits its applications^7,8^. To fulfil the requirement for cathode materials with relatively enhanced properties, an insertion-type cathode of LiNi_1/3_Mn_1/3_Co_1/3_O_2_ composition containing mixed metal ions with relatively higher reversible capacity and better thermal stability is reported for LiBs^6^. It exhibits the similar crystal structure as that of LiCoO_2_ (rhombohedral, *R3m* symmetry, with MO_6_ octahedral sharing the edges (“M” = Co, Ni, and Mn) and Li ions (Li^+^) in the structure occupy the respective octahedral sites.)^9,10^. Ni, Mn, and Co ions exist with valances of +2, +4, and +3, respectively and the Ni^2+^/Ni^4+^ and Co^3+^/Co^4+^ redox couples are electrochemically active during the insertion/removal of Li^+^, whilst the Mn^4+^ ions remain inactive^11–15^. As a consequence, the Mn dissolution and associated Jahn−Teller distortion can be greatly eluded, which in turn results in a more stabilized structure during the electrochemical processes^10,16^. The LiNi_1/3_Mn_1/3_Co_1/3_O_2_ cathode offers a practical achievable specific capacity of above 160 mA h g^−1^ when cycled in 2.5 V to 4.5 V voltage range. This capacity is relatively higher than the LiCoO_2_ (cycled in 3 V to 4.3 V voltage range). Another cathode material investigated for high capacity LiBs is sulfur. Lithium-sulfur batteries (Li-S) are considered as promising for vehicle electrification due to their much higher theoretical specific capacities and energy densities than conventional LiBs. Sulfur is abundantly available in reserves which renders it cost effective and higher electrochemical activity makes it suitable candidate to fit-in as a cathode material for Li-S batteries. However the practical comprehension is still lacking due to various technical challenges, which are more critical than other Li^+^ battery systems. Sulfur although possesses excellent electrochemical activity, the poor electrical conductivity (5 × 10^−31^ S cm^−1^) limits its direct utilization as a positive electrode for practical application^17–22^. To overcome this challenge, sulfur is usually bonded with different conducting hosts by physical adhesion or chemical interactions to provide the missing conductivity to sulfur^23–33^. Second drawback of sulfur utilization is the volume expansion due to series of reactions taking place in Li-S cell during electrochemical process, giving rise to the formation of intermediate products commonly known as Li polysulfide^34–39^. The octa-sulfur (S_8_), an allotrope of elemental sulfur reduces into higher-chain poly-sulfur species (Li_2_S_*x*_, 6 ≤ *x ≤ *8) on reaction with Li^+^ during discharge process^21^. These higher-chain reaction products are soluble in liquid medium and upon dissolution diffuse through the porous separator and reach at the anode region, where they further reduced into lower-chain poly-sulfur species (Li_2_S_x_, 1 ≤ *x* ≤ 2) on reacting with excess Li metal. The to-and-fro shuttling of these intermediate reaction products between the electrodes and inimical reactions taking place at the Li metal anode adversely affect the Li metal surface and result in higher cell resistance, reversible capacity loss and the safety concerns associated with Li dendrite formation and dendrite penetration through separator.

Metallic Li as an anode has been extensively explored for developing the high capacity Li metal batteries due to its exceptionally high theoretical capacity, lower electrochemical potential and light weight^40^. Unlike conventional Li-ion batteries where carbon–based anodes are utilized, which involve the intercalation and removal of Li ions, lithium metal batteries containing Li metal anode involve the direct stripping and plating of Li at the surface of Li metal anode during electrochemical process. The continuous stripping and deposition of Li with extended cycling results in a sphere-like roots of Li^+^ which gives rise to rough surface of Li metal anode and a severe volume change^41–44^. The highly rough surface of Li metal anode in turn results in nonhomogeneous current distribution on the Li nuclei. Gradually, the Li grows in a dendrite on the nuclei and ultimately, these dendrites penetrate through the separator and result in short-circuiting and thermal runaway^45–49^. The affinity of Li to form dendrites with extended cell cycling is highly hazardous and leads to safety concerns in such type of cell. Separators in batteries play a crucial role in regulating the movement of electrolyte matrix containing the mixed ions. Conventionally used polypropylene separators are porous in nature and as-such allow the dendrite penetration and smooth movement of reaction intermediates to travel across the electrodes with extended cell cycling^50^. Extensive research has been devoted towards designing the efficient membrane separators to shield the Li anode and also to anchor the diffusion of polysulfides in Li-S batteries. Various conductive coating materials such as carbon coatings^20^, polymeric coatings^51^, ceramic coatings^52,53^, on separator surface are reported to effectively restrict the polysulfide diffusion during cell cycling. However, these coatings are generally applied on the cathode regions and involve the conventional preparation technique such as slurry casting process which results in higher coating thickness, thus negatively impacting the cell impedance and cell performance due to presence of polymeric binders in the slurry and allowing the Li dendrite penetration due to unprotected Li metal anode. It is therefore essential to address the critical issues associated with the Li metal utilization in Li-S batterers. Magnetron sputtering technique has been highly effective in achieving uniform coating layers of metals/non-metal and their oxides such as Al, Cu, Nb, Pt, Al_2_O_3_, LiNb_2_O_3_ etc., with controlled coating thickness^54–59^. Various metallic nanolayer coatings on to the separator membranes have been reported for mitigation of polysulfide diffusion^60^, as well as supressing the Li dendrite penetration^54,58^. Niobium metal and its oxides possess excellent electrical and mechanical properties and are reported to improve the performance of LiBs and mitigate the serious issues related to polysulfide diffusion in Li-S batteries^61–63^. Recent reports revealed that the oxides of Nb such as Nb_2_O_5_ show strong affinity towards Li polysulfide thereby preventing their dissolution during electrochemical process and effectively improve the battery performance. Herein, we report the niobium metal deposited PP separator prepared via RF magnetron sputtering. High mechanical strength of the Nb coating is advantageous in supporting the structural integrity of Li during the electrochemical process. We demonstrate that this technique can suppress the dendrite penetration by dendrite merging, which is essential for achieving the improved contact and uniform charge distribution at the electrode-electrolyte interface. The nanolayer of Nb metal deposited on one side of the PP separator serves as an additional conducting agent to facilitate electrochemical stripping/deposition of Li metal with relatively very less accumulation of electrically isolated or “dead” Li. Furthermore, the electrically conductive nature of Nb coating guides the deposition of Li metal and controls the Li deposition morphology. The uncoated surface of Nb-PP facing the cathode, serves as an insulating substrate while the conductive side of the Nb-PP separator is in contact with Li anode. The Li symmetric cell with Li||Nb-PP@Nb-PP||Li configuration and Li||Nb-PP||LNMC and Li||Nb-PP||S cells assembled with modified separator membrane display improved stability and better electrochemical performances.

## Results and Discussion

### Microstructure and wettability of PP separator and Nb-PP separator

**Figure 1:**
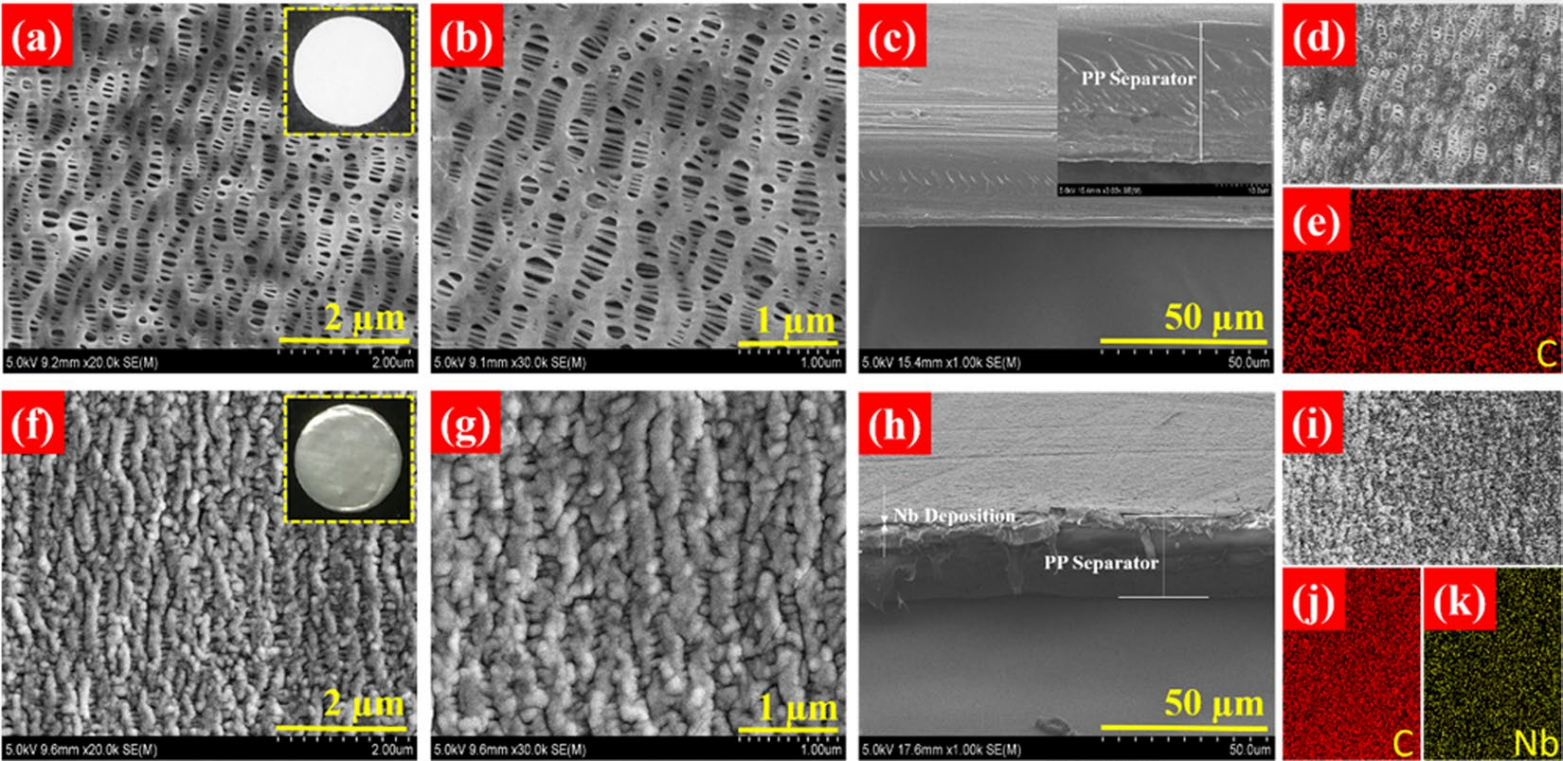
(**a**,**b**) HRSEM images of pristine PP separator and (**c**) it’s cross-sectional view. (**d**,**e**) EDS image and elemental map of PP separator. (**f**,**g**) HRSEM images of Nb-PP separator and (**h**) it’s cross-sectional view. (**i**) EDS image and (**j**,**k**) elemental maps of Nb-PP separator. Insets in (**a**,**f**) are the digital photographs of pristine surface and Nb coated surface of PP separator.

The microstructure of PP separator and Nb-PP separator was studied via scanning electron microscope. Figure 1 displays the SEM micrographs of PP and Nb-PP (with optimized coating thickness of ~60 nm) separators. The SEM images of pristine PP separator display a highly porous and Nb-free surface (Fig. 1a,b). The cross-sectional SEM image of PP separator shows that the thickness of PP was 24.7 µm (Fig. 1c). EDS image and the elemental map confirms the Nb-free surface of porous PP separator (Fig. 1d,e). In contrast, Nb-PP separator displays a uniform and thin deposition of Nb metal particles covering the porous surface of PP separator (Fig. 1f,g). The cross-sectional SEM images of Nb coated PP separator (Fig. 1h) reveals the average Nb coating thickness of 60 nm. EDS image and the elemental maps suggest the homogeneous distribution of Nb deposition onto the PP separator surface (Fig. 1h–k). The EDS data of uncoated surface of Nb-PP separator is presented in Fig. S1. The lightly dense coating on PP separator surface could help in improving the surface wettability towards the liquid electrolyte and protect the separator from Li dendrite penetration, without blocking the channels for Li^+^ transport^56,59^, whilst retaining the good thermal stability. Insets in Fig. 1a,f are the digital photographs of uncoated and Nb coated surfaces of Nb-PP separator. The thermal stability of separators was investigated via DSC and is presented in the Supplementary Information (Fig. S2). For comparison of morphologies of samples, other deposition times were also considered (Fig. S3). For the deposition time of 15 min (Fig. S3a) the coating looks thin with notable porous surface which, in turn could result in homogeneous current distribution at the interface during electrochemical cycling process without impairing the ion transport^56^. For the 30 min (Fig. S3b) deposition time, the surface of separator looks very much flat which may improve the interface contact with Li electrodes but the thick coating may block the Li^+^ during electrochemical process. For the deposition time of 60 min (Fig. S3c), the coating is more obvious and is likely to be fracted onto the PP separator, which could result in uneven Li deposition during galvanostatic cycling process^55,60^. Thus, the sputtering time of 15 min was preferred. The Nb coating on PP separator is expected to assist uniform Li nucleation.

The wettability tests on Nb coated separator were performed via contact angle measurement method with both water and the liquid electrolyte (1.0 M L^−1^ LiTFSI in DME: DOL), respectively. The amount of water and liquid electrolyte utilized for each test was 2 µL. The Nb-PP separator displays a relatively higher wettability towards water and the liquid electrolyte in comparison with the PP separator (Fig. S4). The Nb-PP separator exhibited a contact angle of 70.375° and just 19.25° with water and liquid electrolyte, respectively, while as the PP separator exhibited slightly higher contact angles of 107.09° and 35.56° with water and liquid electrolyte, respectively. The improved wettability of Nb coated separator could help in higher electrolyte uptake which, in turn may result in improved electrochemical performance of a cell.

### Electrochemical testing of symmetric cells

**Figure 2:**
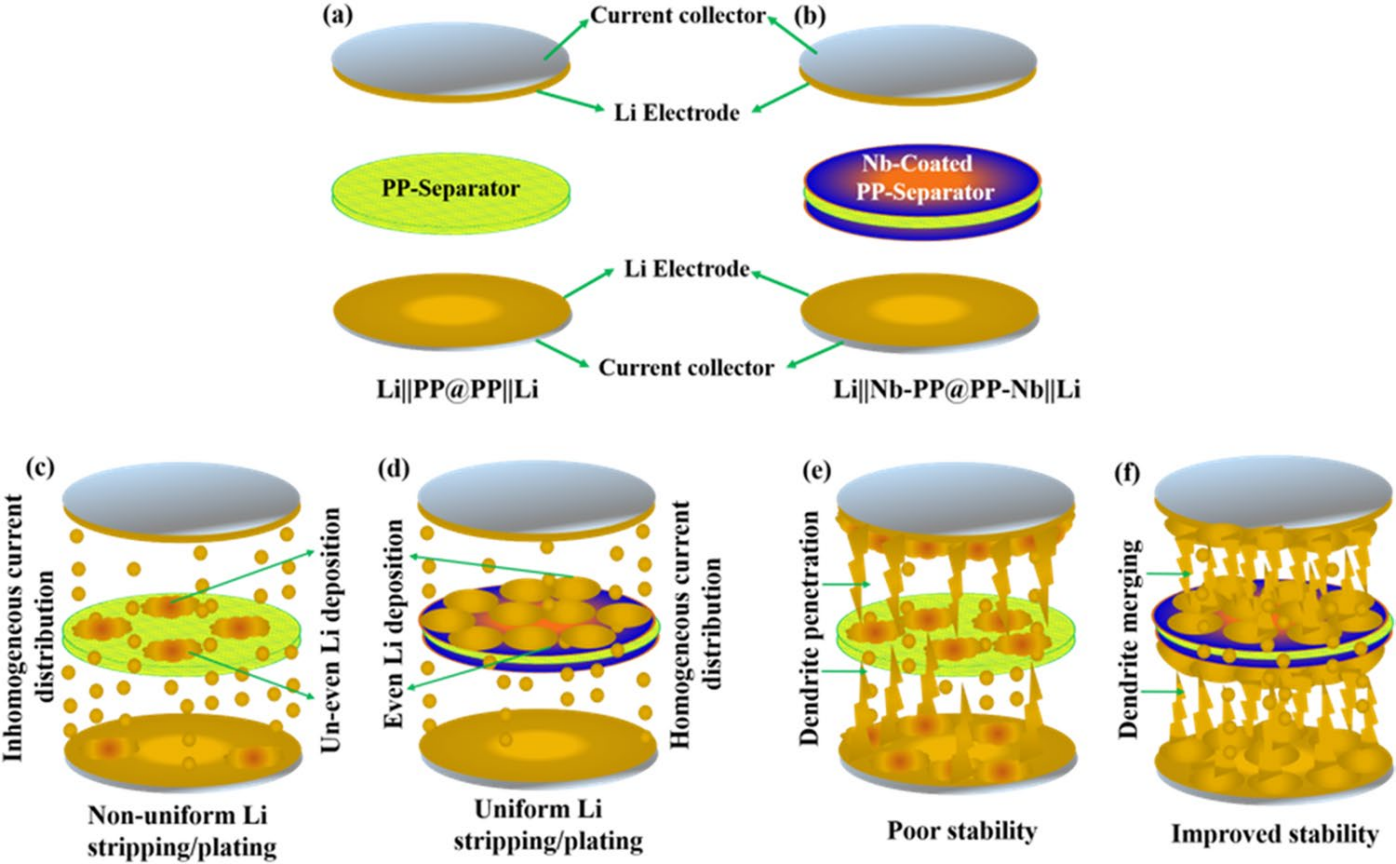
Schematic representation of (**a**) Li||PP@PP||Li symmetric cell and (**b**) Li||Nb-PP@PP-Nb||Li symmetric cell components assembled in Swagelok. (**c**,**d**) Schematic of Li||PP@PP||Li symmetric cell cycling showing uneven and even lithium deposition on to the separators surface. (**e**,**f**) Schematic demonstration of lithium dendrite penetration in Li||PP@PP||Li symmetric cell and lithium dendrite merging in Li||Nb-PP@PP-Nb||Li symmetric cell.

**Figure 3:**
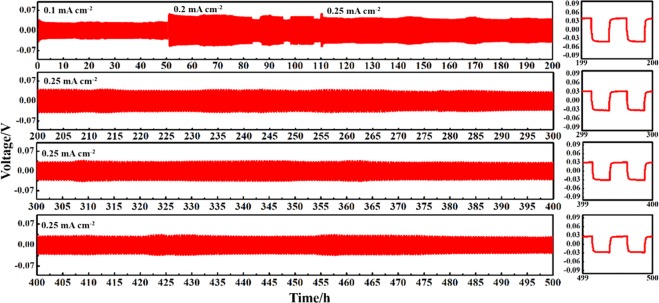
Time dependent voltage profiles of Li||Nb-PP@PP-Nb||Li symmetric cell for 500 h at different current densities reflecting a uniform stripping and deposition of lithium. Plots on the right side present the enlarged view of voltage profiles for the respective cycles.

**Figure 4:**
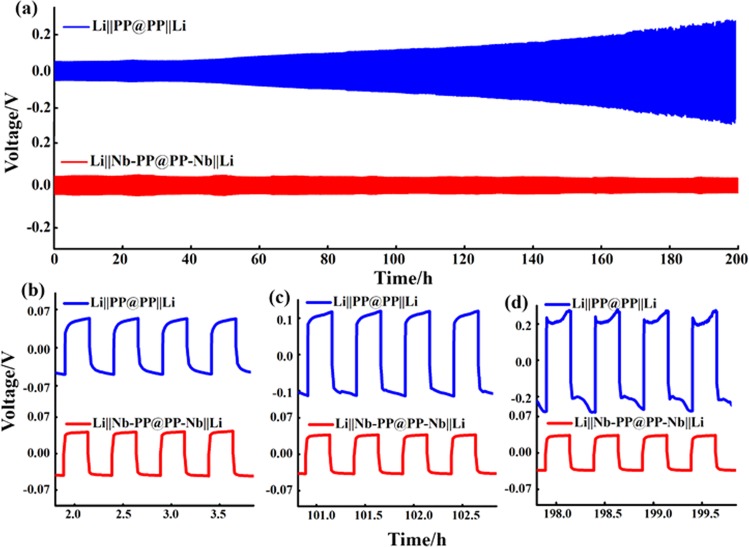
(**a**) Time dependent voltage profiles of Li||PP@PP||Li and Li||Nb-PP@PP-Nb||Li symmetric cells at a current density of 0.5 mA cm^−2^. (**b**–**d**) Enlarged voltage profiles of Li||PP@PP||Li and Li||Nb-PP@PP-Nb||Li symmetric cells for the respective time periods.

**Figure 5:**
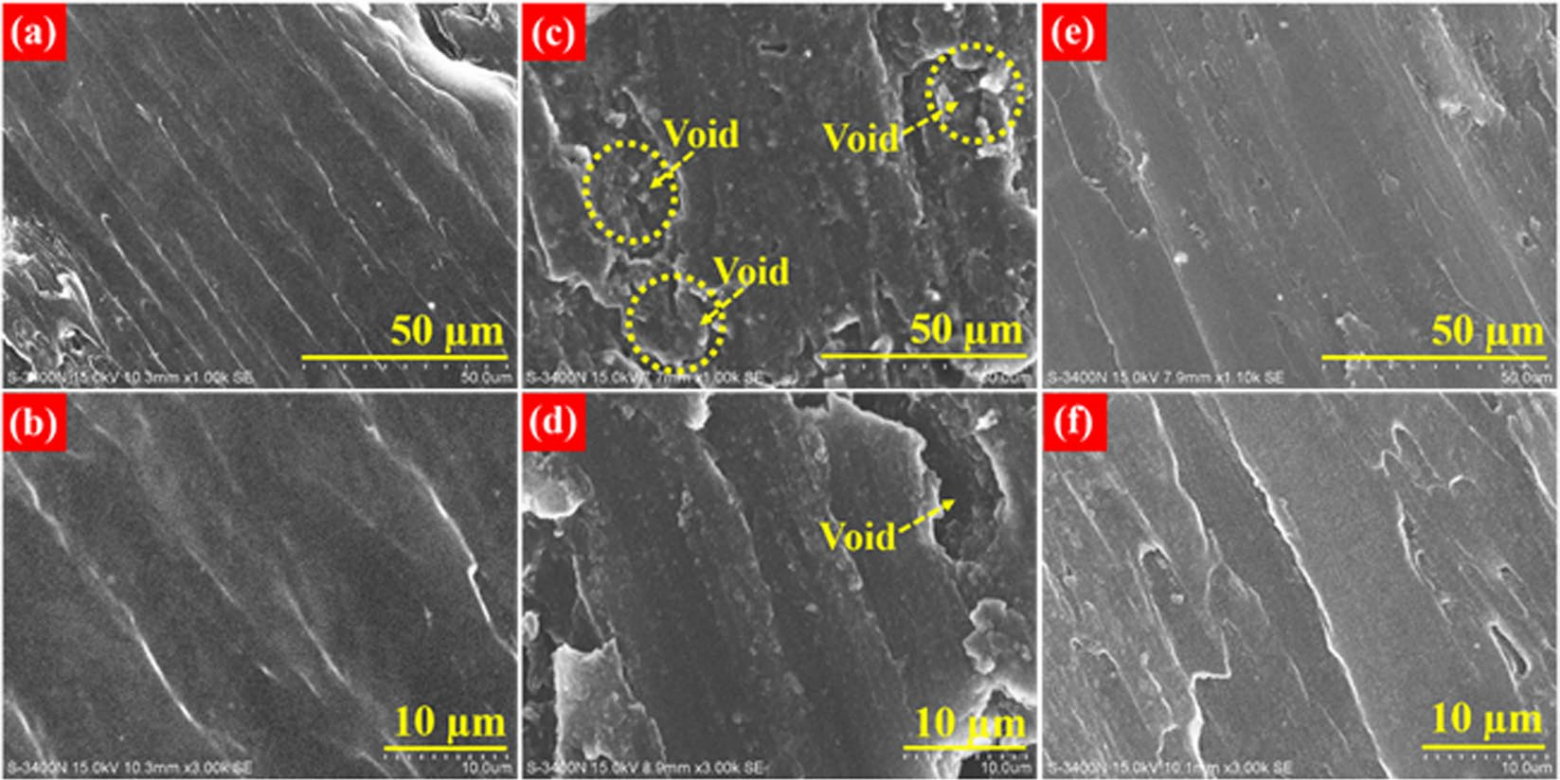
(**a**) SEM image of pristine Li foil revealing a smooth surface. (**b**) SEM image of pristine Li foil at higher magnification. (**c**) SEM image of washed Li foil after cycling with pristine PP separator displaying a rough surface due to inhomogeneous lithium extraction/deposition during galvanostatic cycling. (**d**) It’s magnified view. (**e**) SEM image of washed Li foil after cycling with Nb-PP separator reflecting a relatively smooth surface. (**f**) It’s magnified view.

**Figure 6:**
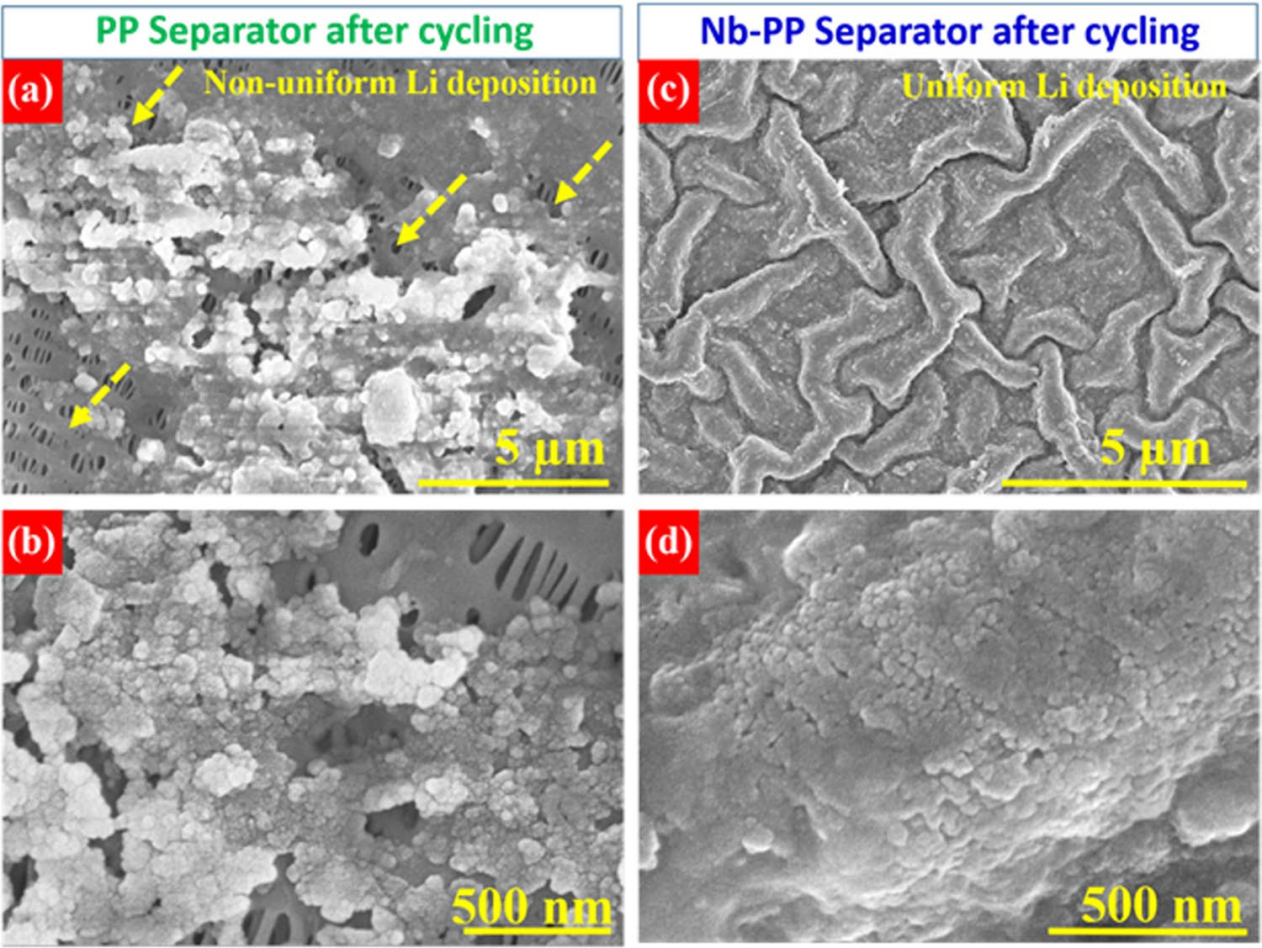
HR-SEM image of (**a**) pristine PP separator surface after cycling (**b**) It’s magnified view. HR SEM image of (**c**) Nb-PP separator surface after cycling and (**d**) It’s magnified view displaying uniform deposition of lithium on the surface of separator.

The schematic illustration of symmetric cell components and the Li stripping/deposition during electrochemical cycling based on PP separator and the Nb-PP separator is presented in Fig. 2(a–f). The electrical property of pristine surface and Nb coated surface of Nb-PP separator was investigated. The uncoated surface of Nb-PP separator was non-conducting when tested with ohmic-meter, indicating no diffusion of Nb coating during sputtering process (Fig. S5a). The Nb coated surface of Nb-PP separator shows surface resistance indicating its conductive nature (Fig. S5b), while as the uncoated surface of Nb-PP separator exhibits highly insulating properties. Galvanostatic cycling was carried out at different current densities in symmetrical Li cell with Li||Nb-PP@PP-Nb||Li configuration to investigate the Li deposition process in this system (Fig. 3). The cycle duration was 30 min and the total time for which the cycling was performed was 500 h. The Li||Nb-PP@PP-Nb||Li symmetric cell exhibited a noise-free voltage profile throughout cycling, suggesting a homogeneous current distribution at the Li metal surface. The symmetric cell exhibited the stable over potential of approximately 0.03 V at 0.1 mA cm^−2^, 0.2 mA cm^−2^, and 0.25 mA cm^−2^, respectively, corresponding to each Li||Nb-PP interface area specific resistance of nearly 30 Ω cm^2^. When cycled at 0.5 mA cm^−2^, the Li||Nb-PP@PP-Nb||Li symmetric cell displayed a relatively lower polarization of 0.04 V with smooth Li stripping and deposition profiles in comparison to Li||PP@PP||Li symmetric cell with huge polarization and erratic Li stripping and plating profile at 0.5 mA cm^−2^ (Fig. 4a). The enlarged voltage profiles of the respective cycles corresponding to Li||PP@PP||Li and Li||Nb-PP@PP-Nb||Li symmetric cells are presented in Fig. 4(b–d). The higher polarization in the Li||PP@PP||Li cell could be associated with the inhomogeneous current distribution at the Li||PP interface because of non-uniform Li deposition on PP separator surface^58^. EIS measurements (Fig. S6) were performed for the symmetric cells to confirm the effect of Nb nanolayer in improving the interface contact. The symmetric cell with PP separator displayed an interface resistance of 125.5 Ω before cycling and an increased contact resistance of 208.2 Ω after cycling (Fig. S6b). The Nb-PP symmetric cell on the other hand, exhibited an interface resistance of 134.5 Ω before cycling and much lower resistance of just 13.8 Ω after cycling (Fig. S6c). Improved interface contact could be the possible reason for reduction in the contact resistance in the latter case. To further confirm the stability of Nb-PP separator a rate performance test for the Li||Nb-PP@PP-Nb||Li symmetric cell was performed at current densities 0.2 mA cm^−2^, 0.4 mA cm^−2^, 0.6 mA cm^−2^, 0.8 mA cm^−2^ and 1 mA cm^−2^, respectively, as depicted in Fig. S7a. The cell exhibited a stable voltage profile at each current density, indicating a uniform current distribution at the interface. EIS tests were performed after every 50 cycles at each current density, reflecting the reduction in interface resistance (Fig. S7b). The R_1_ (electrolyte resistance) values of the cell showed stable area specific resistance of nearly 5.4 Ω cm^2^ before and after cycling for 100 h at 0.2 mA cm^−2^ and 0.8 mA cm^−2^, respectively (see inset in Fig. S7b and Table S1). The relatively stable and lower values of R_1_ and the interface resistance with galvanostatic cycling indicates that the bi-directional growth of Li from the anode surface and the Nb surface could have result in higher stability of the electrode/electrolyte interface^58^. Post-mortem analysis of symmetric cells with PP and Nb-PP separators was performed to investigate the cycled separators and the Li metal surface. The Swagelok-type cells were dissembled inside glove box. The recovered separator and the Li discs were carefully washed with DME to remove the deposited reaction products on the surface and left for drying in glove box. The dried separators and the Li discs were carefully sealed in vacuum boxes for microstructural analysis. The pristine Li displays a smooth surface as depicted in Fig. 5(a,b). The Li surface cycled in pristine PP separator containing symmetric cell exhibits a rough surface with large number of voids created due to inhomogeneous stripping and platting of Li which could have results in non-uniform current distribution at the electrode interface (Fig. 5c,d). The Li surface cycled in Nb-PP separator containing symmetric cell on the other hand exhibited a relatively smoother surface without the presence of any visible voids (Fig. 5e,f). The improved stability behaviour of Li surface could be ascribed to bi-directional growth of Li on anode and Nb surface, resulting in homogenous stripping and platting of Li during galvanostatic cycling^46,56,58,64^. The Li deposited on the surface of Li anode grows towards the Nb surface of PP separator and the Li deposited on Nb-PP surface of PP separator grows towards the Li anode. The grown Li from two ends merge together and results in not only the uniform current distribution but also prevents the Li dendrites to cross the PP separator which otherwise could result in thermal runaway due to short circuiting^56^. Besides, the HRSEM analysis of cycled PP separator and the Nb-PP separator was performed to investigate the nature of deposition of Li onto the separators surface. The PP separator surface displayed an un-even deposition of Li (Fig. 6a,b), which could have resulted in the non-uniform current distribution and finally the increased polarization voltage in the symmetric cell with Li||PP@PP||Li configuration. The SEM images of cycled separators and Li surface displaying the morphology of lithium deposition are presented in Figs. S8 and S9, respectively. EDS image of cycled PP separator and the associated elemental maps are presented in Fig. S10 in the supplementary information. HRSEM images of cycled Nb-PP separator on the other hand display highly uniform deposition of Li on the separator surface (Fig. 6c,d), which in turn could have resulted in the homogeneous current distribution across the interface and hence the stable polarization voltage. EDS image of cycled Nb-PP separator further confirm the uniform deposition and stripping of Li metal responsible for homogeneous current distribution across the interface (Fig. S11). The above result confirms the effect of Nb coating layer in improving the electrochemical stability of the Li symmetric cell and is in well, agreement with the reported literature^56,58^.

### Electrochemical performance evaluation of half cells containing LNMC cathode and composite sulfur cathode

**Figure 7:**
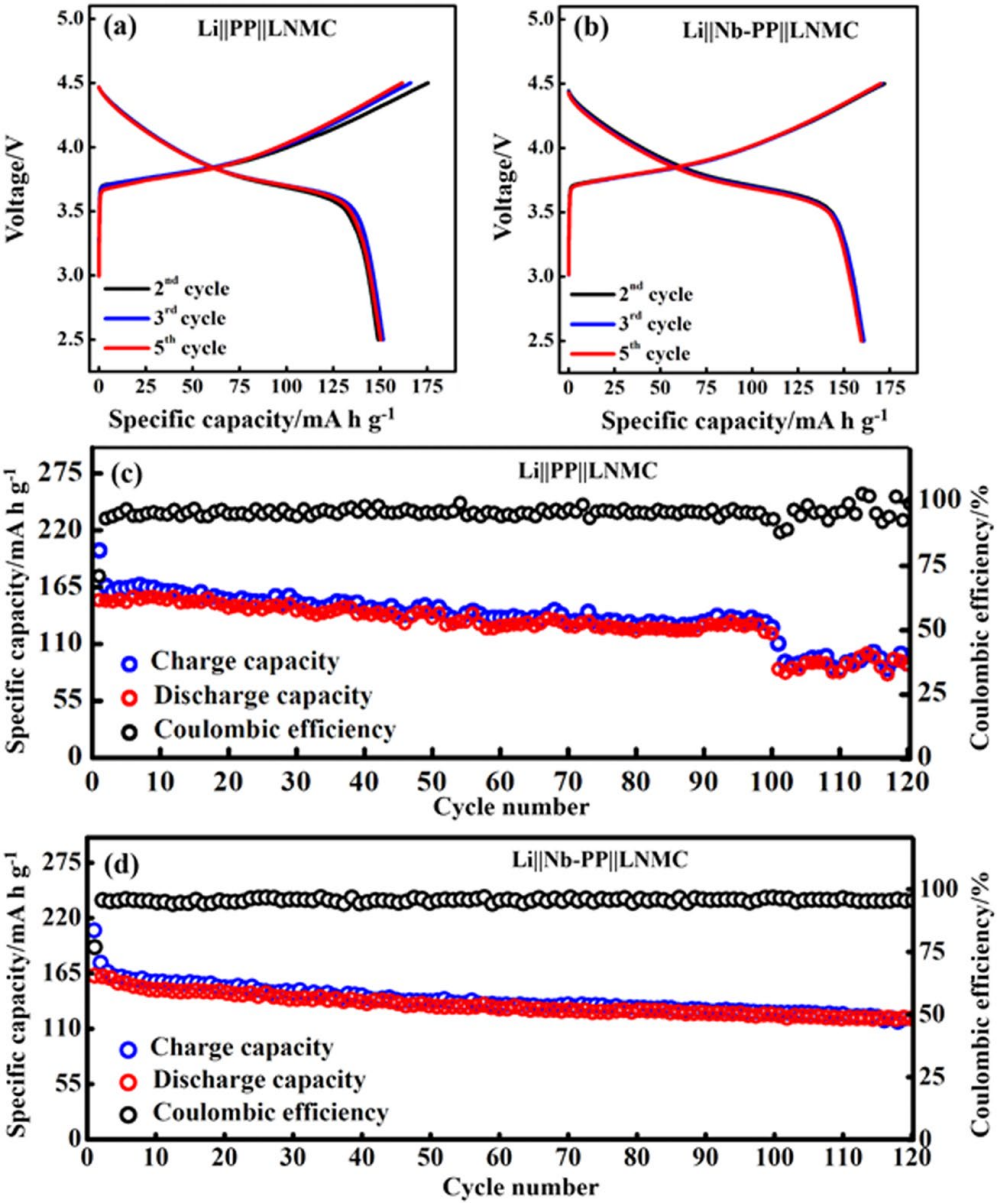
(**a**) Voltage profiles of LNMC cathode cell with PP separator. (**b**) Voltage profiles of LNMC cathode cell with Nb-PP separator. (**c**) Charge-discharge capacity and Coulombic efficiency of LNMC cathode PP separator. (**d**) Charge-discharge capacity and Coulombic efficiency of LNMC cathode with Nb-PP separator showing improved cycling stability.

To investigate the effectiveness of Nb nanolayer deposition on cell performance, cells with LNMC and composite sulfur cathodes were constructed. CV measurements were performed for the LNMC cathode based cells in 2.5 V to 4.5 V voltage range at 0.1 mVs^−1^. Cells containing PP and Nb-PP separators show a pair of redox peaks corresponding to the Ni^2+^/Ni^4+^ during the Li^+^ insertion and removal^16^. The redox peaks for the first CV scan are centered at 4.16 V and 3.65 V respectively, resulting in a potential difference of 0.51 V (Fig. S12). The potential difference shows a dip after successive scans and attained a minimum value of 0.17 V for the 10^th^ CV scan attaining a peak current of magnitude 0.10 mA. The Nb-PP separator based cell on the other hand, displayed the potential difference of only 0.15 V corresponding to the redox peaks at 4.14 V and 3.63 V for the first CV scan with reduced potential difference value of 0.19 V and higher peak current of magnitude 0.13 mA. The exceptionally low potential difference indicates the better cycling reversibility^16^. The relatively higher peak current in the latter case could be associated with the improved electrical contact between Nb and Li metal anode which may promote fast Li^+^ transport across the anode|electrolyte interface^65^. These results are in accordance with the Randles-Sevcik equation, where peak current (I_p_) is proportional to the square root of diffusion coefficient (D_Li_^+^) of Li^+^. Furthermore no reduction peak near 3.0 V was observed in the CV curves and it can be concluded that Mn retained its +4 oxidation status in the structure. The Mn^4+^ ions are inactive within the LNMC lattice, as a consequence it results in stabilizing the lattice. In the CV cycle a homologous integral area was observed, suggesting that the Li intercalation and intercalation was highly reversible^10^. Figure 7(a,b) show the charge-discharge curves of LNMC cells without and with Nb-PP separator in a stable voltage range of 2.5 V to 4.5 V. The corresponding specific capacities and efficiencies with cycle number are presented in Fig. 7(c,d). The PP separator based LNMC cell delivered an initial discharge capacity of 150 mA h g^−1^ and a discharge capacity of 90 mA h g^−1^ after 120 cycles at 0.2 C (1 C = 180 mA h g^−1^) with Coulombic efficiency of below 95%. In contrast, the charge-discharge curves corresponding to Nb-PP show better reversibility with an initial discharge capacity of 165 mA h g^−1^ at 0.2 C. The cell exhibited reasonable reversible capacity of 130 mA h g^−1^ for the 120^th^ discharge with an efficiency of above 97%. The relatively better cycling performance of LMNC cell with Nb-PP separator could be ascribed to the improved Li^+^ diffusion at the electrode electrolyte interface and better stability of Li metal anode during electrochemical process. The above results suggest that the Nb coating on PP separator not only allows enhanced Li^+^ transport but also acts as a buffer for the Li dendrites growth and penetration.

**Figure 8:**
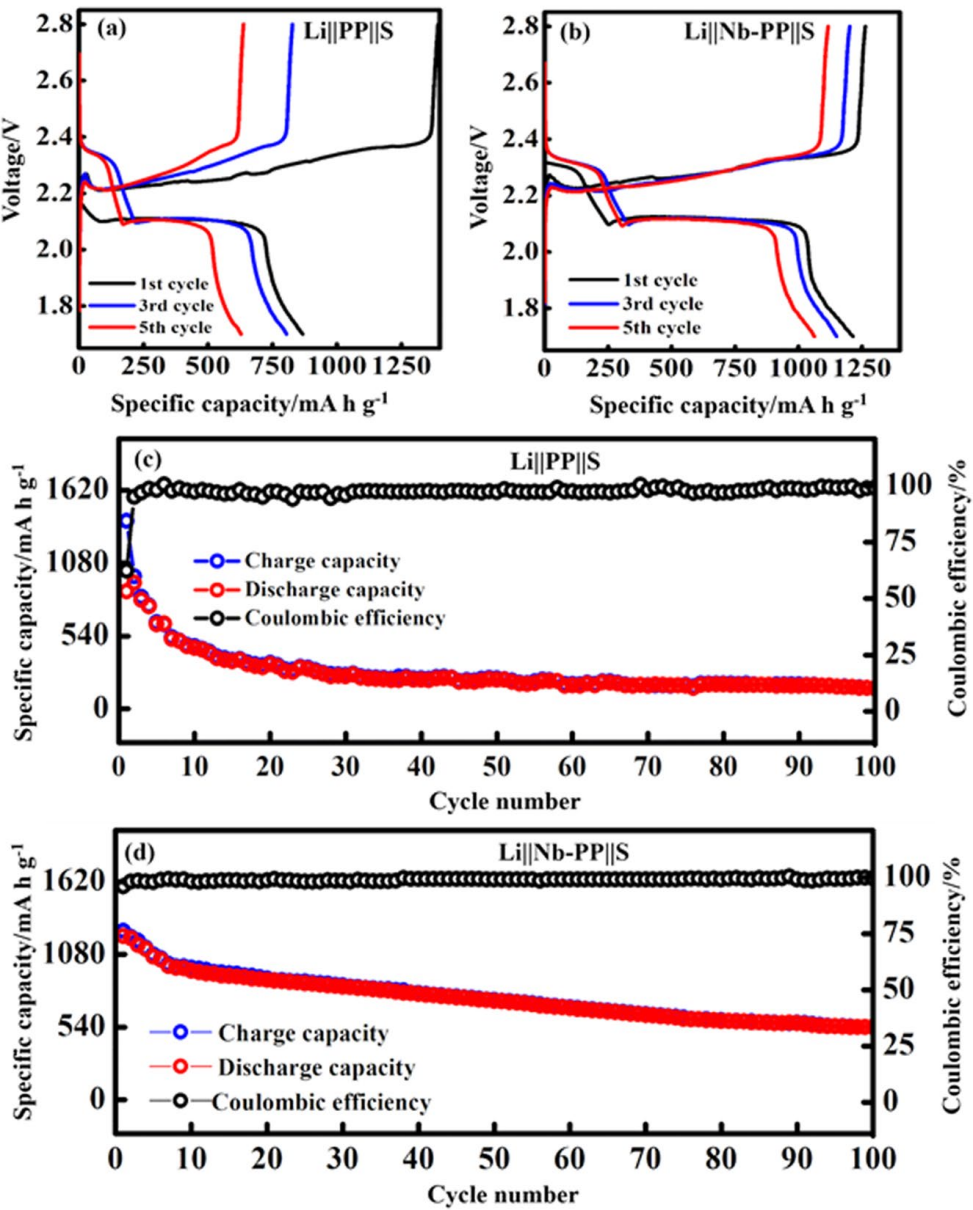
(**a**) Voltage profiles of Li-S cell with PP separator. (**b**) Voltage profiles of Li-S cell with Nb-PP separator. (**c**) Charge-discharge capacity and Coulombic efficiency of PP separator showing fast capacity decay. (**d**) Charge-discharge capacity and Coulombic efficiency of Nb-PP separator showing improved capacity retention.

As mentioned earlier, the exceptionally higher theoretical capacity of sulfur makes it an ideal material for Li metal batteries with higher energy densities. Li-S cells were constructed to demonstrate the effect of Nb coating on supressing the Li dendrite penetration and the polysulfide anchoring. The CV test in 1.7 V to 2.8 V voltage range at 0.1 mV s^−1^, was conducted for the Li-S cells containing pristine PP and Nb-PP separators (Fig. S13a,b). Two reduction peaks were observed in the cathodic scan at around the potential of 2.25 V and 2.01 V corresponding to the reduction of sulfur to long-chain polysulfides (Li_2_S_n_; 4 ≤ *n ≤ *8) and at 2.0 V corresponding to the further reduction of long-chain Li polysulfide into insoluble short- chain Li polysulfides. In the anodic scan, two oxidation peaks observed at 2.34 V and 2.39 V are associated with the conversion of lower order Li polysulfides back to elemental sulfur^66–68^. For the Li-S cell with PP separator, the CV curves at higher potential (2.5 V to 2.75 V) in the anodic region look very much noisy with small humps (see inset in Fig. S13a) which could be associated with the possible Li polysulfide diffusion. Also the area under CV curves decreases with successive CV scans, indicating sluggish conversion kinetics and the capacity fade due to poor reaction reversibility^69,70^. Li-S cell with Nb-PP separator exhibited stable and smooth CV curves in the higher potential (see inset in Fig. S13b) with relatively similar area under the curves, indicating better stability and cycling reversibility. To further demonstrate the electrochemical stability galvanostatic cell cycling in 1.7 V to 2.8 V voltage range at room temperature (25 °C) was performed for Li-S cells containing PP and Nb-PP separator. Figure 8(a,b) represents the voltage profiles of Li-S cell constructed with PP and Nb-PP separators. The Li-S cell PP separator delivered the first discharge capacity of 867 mA h g^−1^ and a charge capacity of 1393 mA h g^−1^ at 0.1 C (1 C = 1672 mAh g^−1^). A low discharge capacity of just 151 mA h g^−1^ after 100^th^ discharge cycle was retained by the Li-S cell with PP separator. The fast capacity fade could be associated with the highly porous structure of PP separator which is permeable to Li polysulfides and the Li dendrites^71,72^. It is worth mentioning that the extended voltage profile is observed during initial charging cycle in Li-S cell with pristine PP separator triggering longer charge times before attaining the upper cut off voltage, which could possibly be associated either with the Li polysulfide shuttling phenomenon during charging process^73^ or with the lower open circuit voltage of the cell^74^ (lower discharge capacity was observed for the initial cycle). In contrast, the Li-S cell with Nb-PP separator delivered a first discharge capacity of 1214 mA h g^−1^ and a charge capacity of 1264 mA h g^−1^ at 0.1 C with no extending voltage profile, indicating better stability due to Li dendrite prevention and possibly the polysulfides anchoring effect of Nb-PP separator. The above results are in well agreement with the CV data presented in Fig. S13. An improved discharge capacity of above 540 mA h g^−1^ after 100^th^ discharge cycle was retained by the Li-S cell containing Nb-PP separator. The specific capacities and the corresponding Coulombic efficiency for the respective cells with cycle number are displayed in Fig. 8(c,d). It is clear that the Li-S constructed with PP separator displayed a sharp decrease in the capacity in the initial cycles and finally attaining a lower values of stable specific capacity after 100 cycles with an overall efficiency of less than 97%. The huge capacity fade in conventional Li-S cell may be ascribed to the diffusion of Li polysulfides through porous PP separator, leading to the active material loss and the inimical reactions taking place at the Li anode resulting in corrosion of Li metal and the electrolyte exhaustion^71^. The Li-S cell containing Nb-PP separator exhibited a relatively stable cyclic behaviour and good capacity retention after 100 galvanostatic cycles with nearly 100% cycling efficiency. EIS measurements of the fabricated Li-LMNC and Li-S cells were performed to further confirm the effect of Nb layer on the Li^+^ diffusion kinetics (Fig. S14a,b). The as-assembled Li-LNMC and Li-S cells with pristine PP separator exhibited high interface resistances of 534 Ω and 255 Ω, respectively, which could be associated with the poor contact at the electrode interface^75,76^. In contrast, the as-assembled Li-LNMC and Li-S cells with Nb-PP separator displayed relatively lower values of interface resistances of 202 Ω and 67.95 Ω, respectively. The lower resistance in the latter case is the outcome of improve contact at the anode electrolyte interface because of Nb nanolayer, which could allow the fast Li^+^ diffusion and reduce the charge transfer resistance. These results are consistent with the CV and galvanostatic charge-discharge cycling measurements, where Nb-PP based cell exhibited higher performances.

## Conclusion

We report binder-free Nb metal nanolayer coated PP separator via RF magnetron sputtering for high capacity Li metal batteries. The Nb metal nanolayer modified PP separator exhibited improved hydrophilic character which in turn resulted in its improved wettability with liquid electrolyte. Li symmetric cells constructed with Nb metal nanolayer deposited PP separator exhibited stable voltage profiles and delivered improved cycling performances at various current densities. The improved electrochemical characteristics of Nb-PP separator could be ascribed to the electrical properties of Nb nanolayer whilst its interaction with metallic Li allow bi-directional growth of lithium to prevent the Li dendrite penetration through PP separator. The Nb and Li metal interactions at the interface of the cell resulted in uniform stripping and deposition of Li which in turn promote the homogeneous current distribution. The half-cells of LNMC and sulfur cathodes constructed with modified PP separator delivered an improved electrochemical cycling performances at room temperature. The Nb coating on PP separator greatly improves the battery safety by supressing the Li dendrite penetration. This study could pave the way for design and construction of multifunctional separators for high capacity and safe battery operations.

## Materials and Methods

Commercially available Polypropylene separator (Celgard 2325, 25 µm thickness), niobium (Nb) metal target (99.995% purity and 2 ˝diameter), Ar gas (99.99% purity), and LiNi_0.33_Mn_0.33_Co_0.33_O_2_ (LNMC, Sigma Aldrich), sulfur powder (99.99%, Alfa Aesar), lithium metal (99.99%, Alfa Aesar), LiPF_6_ in DMC (Alfa Aesar), LiTFSI (99.9%, Sigma Aldrich), DME (99.5%, Sigma Aldrich), DOL (99%, Sigma Aldrich), LiNO_3_ (99.99%, Sigma Aldrich) were purchased and used without further purification unless specified.

### Preparation of Nb coated polypropylene separator

Niobium metal coating on to the polypropylene (PP) separator was performed at room temperature (25 °C) using radio frequency (RF) magnetron sputtering technique. PP separator (Celgard 2325) sheet of 3 × 3 cm size was carefully cleaned with ethanol and dried in vacuum overnight. After drying the PP separator was placed into the sputtering chamber (APT Global India). Niobium (Nb) metal (99.995 purity) disc of 2″ diameter was used as a coating target. The distance of 25 cm was fixed between the target and substrate to avoid any physical damage to PP separator during the deposition processes. High purity (99.995%) argon (Ar) gas was utilized as a carrier gas to achieve the Nb deposition. A high vacuum of 5 × 10^−6^ mbar was generated and a chamber pressure was maintained at 5 × 10^−3^ mbar. Initially the RF power of 30 W was applied for 30 min to eliminate any possible contamination from the Nb target surface. Later the RF power of 25 W was supplied to achieve the Nb deposition under Ar flow at low pressure and a controlled Ar gas flow rate of 20 cm^3^/min using a pneumatic mass flow control integrated with the RF system. The total deposition time was 15 min. Deposition of Nb on PP separator was also carried out for 30 min and 60 min of time for comparison. After completing the deposition process the coated PP separator was transferred to glove box to avoid any surface contamination.

### Preparation of LNMC cathode and sulfur cathode

For the preparation of LNMC cathode, a composition of ternary LNMC materials LiNi_0.33_Co_0.33_Mn_0.33_O_2_: Poly (vinylidene fluoride) (PVDF) dissolved in 2, 2-N-methyl-pyrolidinone (NMP): acetylene black (AB) in 8:1:1 weight ratio was finely blended to form a slurry. The slurry was uniformly spread onto the aluminium tape using doctor blade. The coated aluminium tape was transferred to vacuum oven and heated at 120 °C for 24 h. After drying the tape was punched into small discs and transferred to Ar-filled glove box for cell assembling.

The elemental sulfur and active carbon in a weight ratio of 1:1 were hand mixed in agate motor and later placed in a sealed glass bottle under Ar atmosphere. The glass bottle was heat treated in vacuum at 155 °C for 15 h and later slowly cooled down to room temperature to obtain the carbon-sulfur composite.

For the composite cathode preparation the carbon-sulfur composite was mixed with PVDF dissolved in NMP and AB in a weight ratio of 8:1:1 to obtain a slurry. The slurry was blade coated on to the Al tape. The coated Al tape was vacuum dried at 60 °C before shifting to the glove box for cell assembling.

### Materials characterization

Scanning electron microscopy (SEM) was utilized to view the microstructure and cross-section of PP separator, Nb-PP separator, pristine and cycled Li metal surface. To avoid any possible effect of coating layers, Li and Nb-PP samples were sputter coated without any additional carbon (C) or gold (Au). Energy X-ray dispersive spectroscopy (EDS) analysis of Nb coated PP separator was carried out for elemental analysis. Contact angle measurements were performed on the Nb-PP separator with water and liquid electrolyte to analyse the wettability of the modified separator. Differential scanning calorimetry was performed to investigate the thermal stability of Nb-PP separator.

### Electrolyte preparation, cell assembling and electrochemical characterization

Ether based organic liquid electrolyte containing 1.0 M L^−1^ lithium bis(trifluoromethane) sulphonimide (1.0 M L^−1^ LiTFSI) in mixed solvents of 1,2-dimethoxyethane (DME) and 1,3-dioxolane (DOL) (V/V = 1:1) was prepared inside Ar-filled glove box. Briefly, the stoichiometric amount of LiTFSI salt was first dried at 100 °C inside glove box. After drying, it was weighted for 1.0 M L^−1^ and transferred into a sealed glass bottle. DME and DOL solvents were then added in equal volume ratios (1:1 volume ratio) to LiTFSI salt to prepare the electrolyte solution. The LiTFSI salt was dissolved in solvents under continuous magnetic stirring for 24 h. Cell assembling was performed inside Ar-filled glove box (H_2_O and O_2_ ≤ 0.5 ppm). Symmetric cells of Li||PP@PP||Li and Li||Nb-PP@PP-Nb||Li configuration with Li metal discs of area 0.5 cm^2^ and two PP separators/Nb-PP separators (hereafter PP@PP/Nb-PP@PP-Nb) of area 1.13 cm^2^ sandwiched together with Nb coated side facing towards lithium metal in the later configuration were fabricated inside glove box. 30 µL of 1.0 M L^−l^ LiTFSI in DME: DOL was used as liquid electrolyte in each symmetric cell.

LNMC cathode based cells were fabricated in CR 2032 configuration containing a LNMC cathode disc (2.5 mg_LNMC_ cm^−2^) of area 0.785 cm^2^, well cleaned Li disc of area 1.13 cm^2^ as reference and counter electrode, 2 Nb–PP separator with Nb coated side facing towards Li and 25 µL of 1.0 M L^−1^ lithium hexafluorophosphate (LiPF_6_) dissolved in dimethyl carbonate (DMC) as a liquid electrolyte. Composite sulfur cathode based cells were fabricated in CR 2032 configuration containing a sulfur cathode disc (2.0 mg_sulfur_ cm^−2^) of area 0.785 cm^2^, Li disc of area 1.13 cm^2^, Nb-PP separator with Nb coated side facing towards Li and 25 µL of 1.0 M L^−1^ LiTFSI containing 0.1 M L^−1^ LiNO_3_ in DME and DOL (V/V = 1:1). The assembled cells were aged for 24 h before the electrochemical measurements. All electrochemical measurements were performed under room temperature conditions (25 °C). Electrochemical impedance spectroscopy (EIS) tests and cyclic voltammetry (CV) were carried out in Bio Logic VSP-300 workstation. Cell cycling measurements were performed in Bio Logic VSP-300 and Arbin battery testers.

## Supplementary information

Supplementary information
