# Supplementary material for: Metal Coated Polypropylene Separator with Enhanced Surface Wettability for High Capacity Lithium Metal Batteries
Source: Sci Rep. 2019 Nov 14;9:16795. doi: 10.1038/s41598-019-53257-4 (PMC6856152; doi:10.1038/s41598-019-53257-4)
Supplement: Supplementary file 1 — Supplementary information [file 41598_2019_53257_MOESM1_ESM.pdf]

Supplimentary Information

Metal Coated Polypropylene Separator with Enhanced Surface Wettability for High Capacity Lithium Metal Batteries

Mir Mehraj Ud Din and Ramaswamy Murugan*

High Energy Density Batteries Research Laboratory, Department of Physics, Pondicherry University, Puducherry-605014, India

*Email. moranamurugan.phy@pondiuni.edu.in

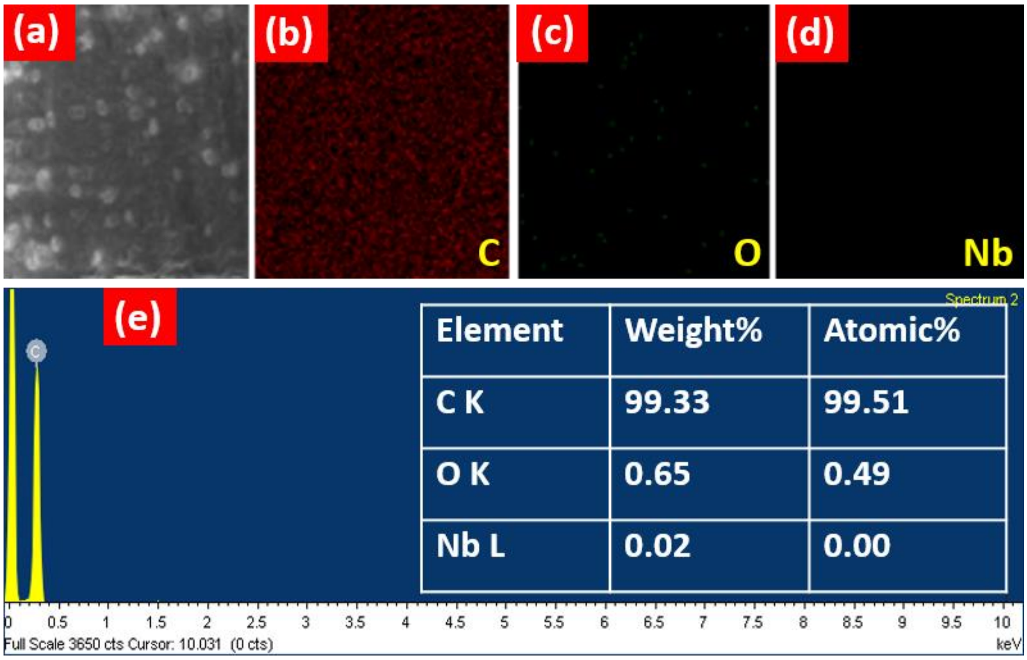

Fig. S1. EDS image of (a) uncoated side of Nb-PP separator and (b,c) its corresponding elemental maps and ED spectrum of Nb-PP. The atomic% of the corresponding elements is given in the table (inset in e).

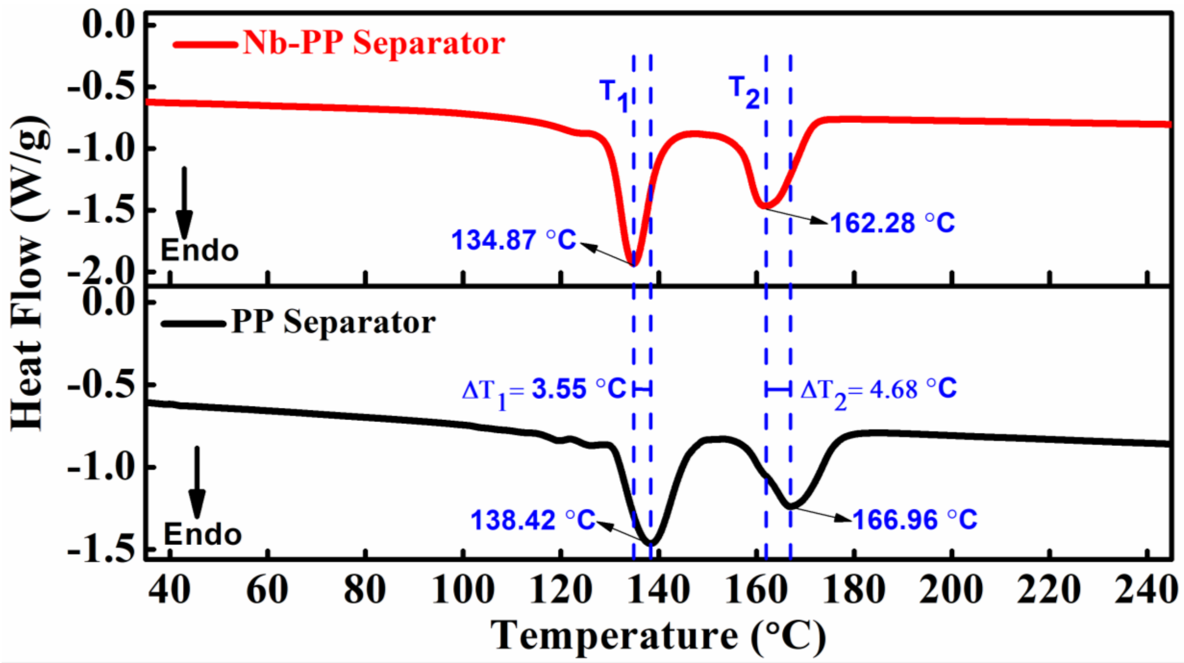

Fig. S2. Differential scanning calorimetry measurements of tri-layer (Celgard 2325) PP separator and Nb-PP separator displaying a relatively similar melting temperatures. The peak at 134.42/134.87 ºC is assigned to the melting of polyethylene layer and the peak at 166.96/162.28 ºC is assigned to the melting of polypropylene layers.

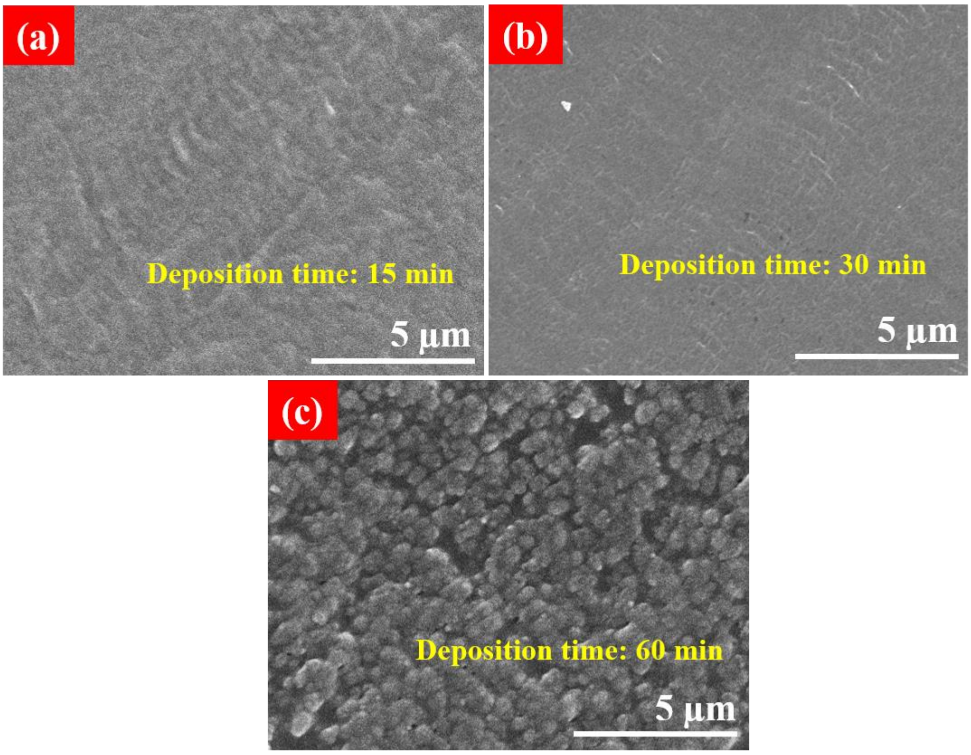

Fig. S3. SEM images of niobium metal coated polypropylene separators with different deposition times.

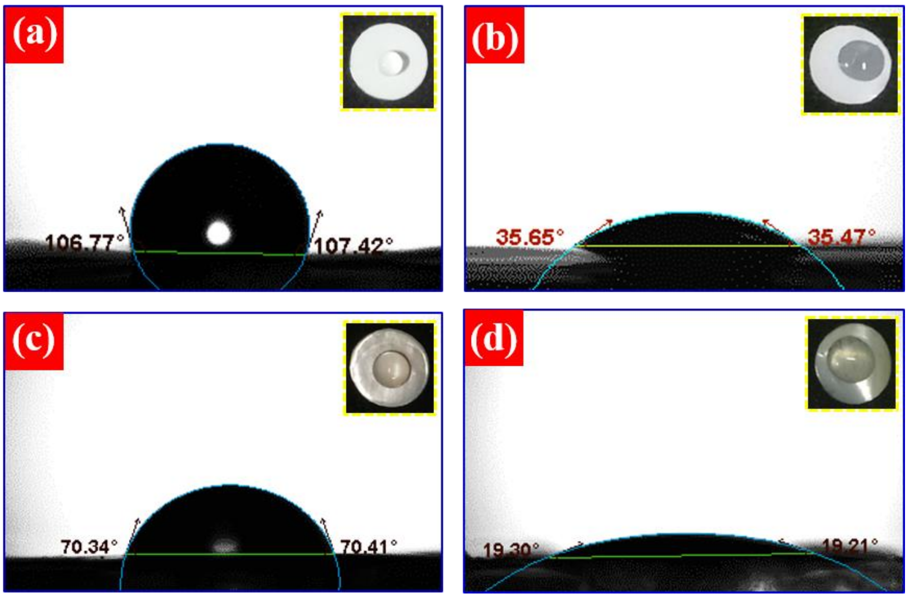

Fig. S4. Contact angle of pristine PP separator with (a) water and (b) liquid electrolyte. Insets in (a) and (b) are the digital photographs of pristine PP separator surface wetted with a water and liquid electrolyte drop, respectively, displacing poor wettability. Contact angle of Nb-PP separator with (c) water and (d) liquid electrolyte. Insets in (c) and (d) are the digital photographs of Nb-PP separator surface wetted with a water and liquid electrolyte drop, respectively, displaying improved wettability.

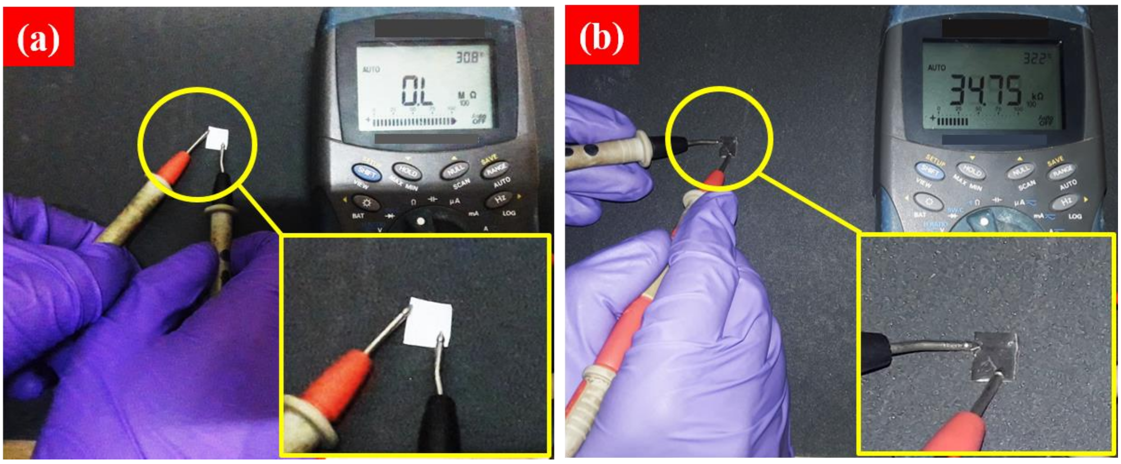

Fig. S5. Measurement of surface resistivity using ohmic meter for both sides of separator surfaces; (a) uncoated surface of Nb-PP separator. (b) Nb coated surface of Nb-PP separator.

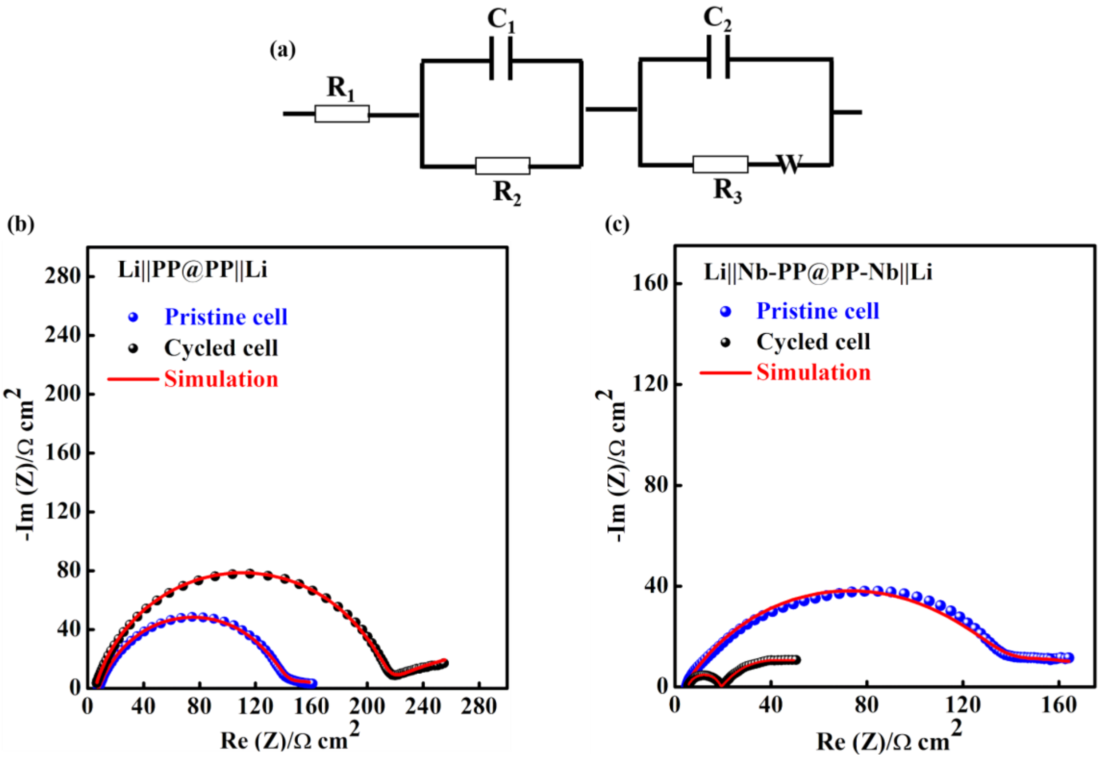

Fig. S6. (a) Circuit diagram for simulation of EIS data. EIS plots of (b) Li||PP@PP||Li and (c) Li||Nb-PP@PP-Nb||Li symmetric cells in 2 MHz to 0.01 Hz frequency range and 10 mV ac amplitude before and after galvanostatic cycling. Insets represents the EIS of respective symmetric cell configurations in high frequency region.

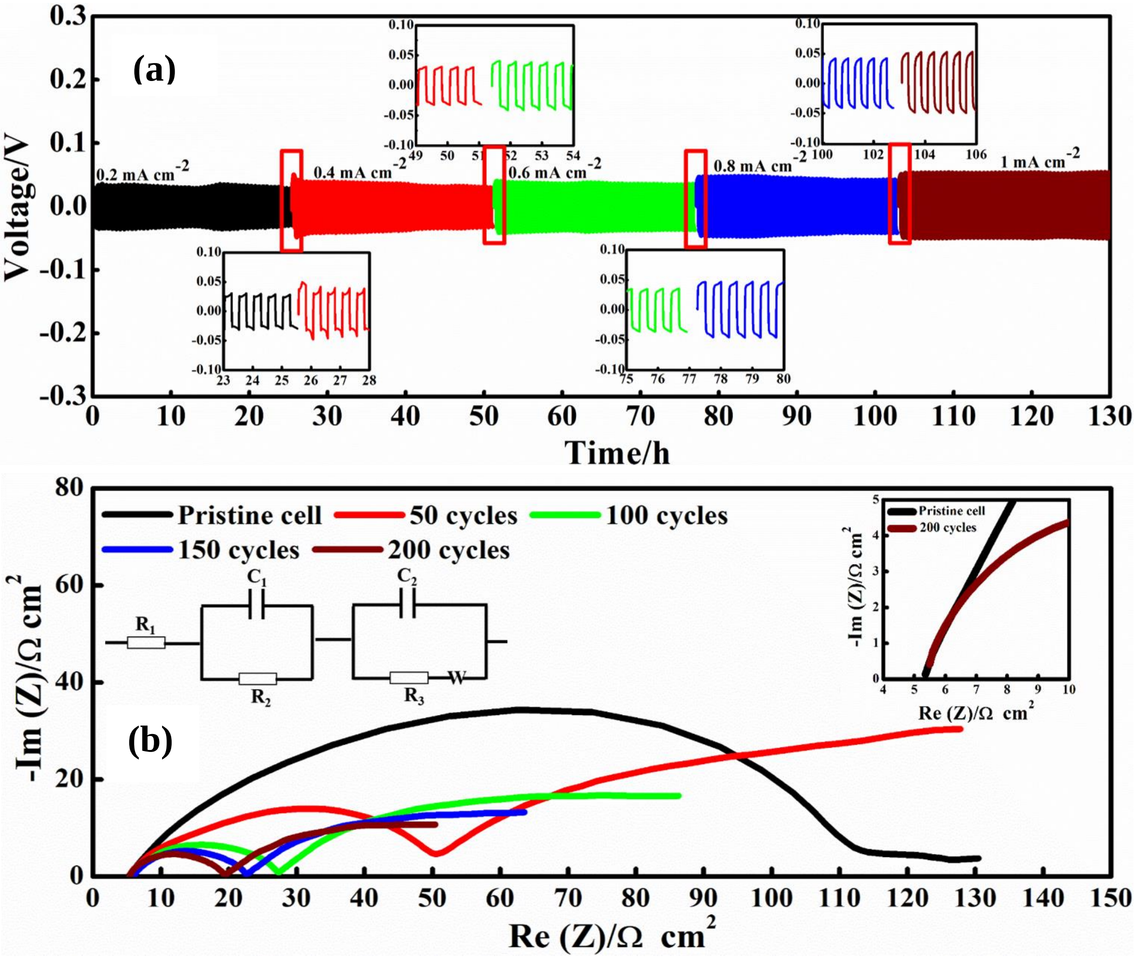

Fig. S7. (a) Time dependent voltage profile of Li||Nb-PP@PP-Nb||Li symmetric cell at higher current densities displaying uniform stripping and plating of lithium at highest current density of 1.0 mA cm^{-2}. Insets represent the enlarged voltage profiles for the respective time periods. (b) EIS of Li||Nb-PP@PP-Nb||Li symmetric cell in a frequency range of 2 MHz to 0.01 Hz before cycling and after every 50 cycles. Insets represent the circuit diagram and EIS of Li||Nb-PP@PP-Nb||Li symmetric cell in high frequency region before cycling and after 200 cycles with nearly equal values of R_1. The parameters R_1, R_2 and R_3 correspond to the bulk resistance, the interfacial resistance and charge transference resistance, respectively. C and W represents a constant phase element and Warburg element, respectively.

Table S1.

	Parameters		
	R_1 (Ω cm^2)	R_2 (Ω cm^2)	R_3 (Ω cm^2)
**Before cycling**	5.35	-	125.1
**After 50 cycles at 0.2 mA cm^{-2}**	5.6	44.9	82.9
**After 100 cycles at 0.4 mA cm^{-2}**	5.7	21.6	64.6
**After 150 cycles at 0.6 mA cm^{-2}**	5.75	16.85	46.65
**After 200 cycles at 0.8 mA cm^{-2}**	5.4	14	36.4

Table S1. Resistance values of Nb-PP separator based Li||Li symmetric cells deduced from the impedance plots corresponding to various current densities. The parameters R_1, R_2 and R_3, respectively, represent the bulk resistance associated with electrolyte, contact resistance at electrolyte/electrode interface and the charge transfer resistance associated with Li^+ diffusion.

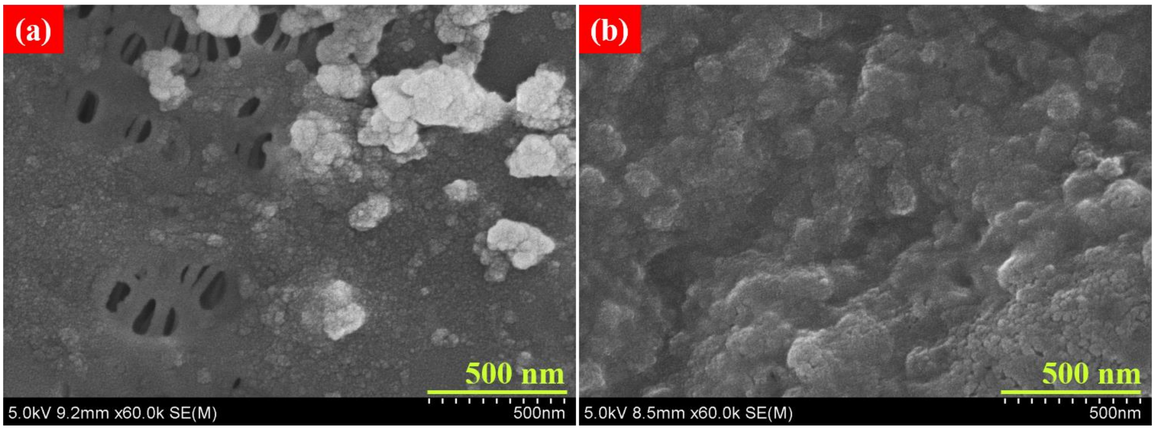

Fig S8. HRSEM images of (a) PP separator and (b) Nb-PP separator after cycling.

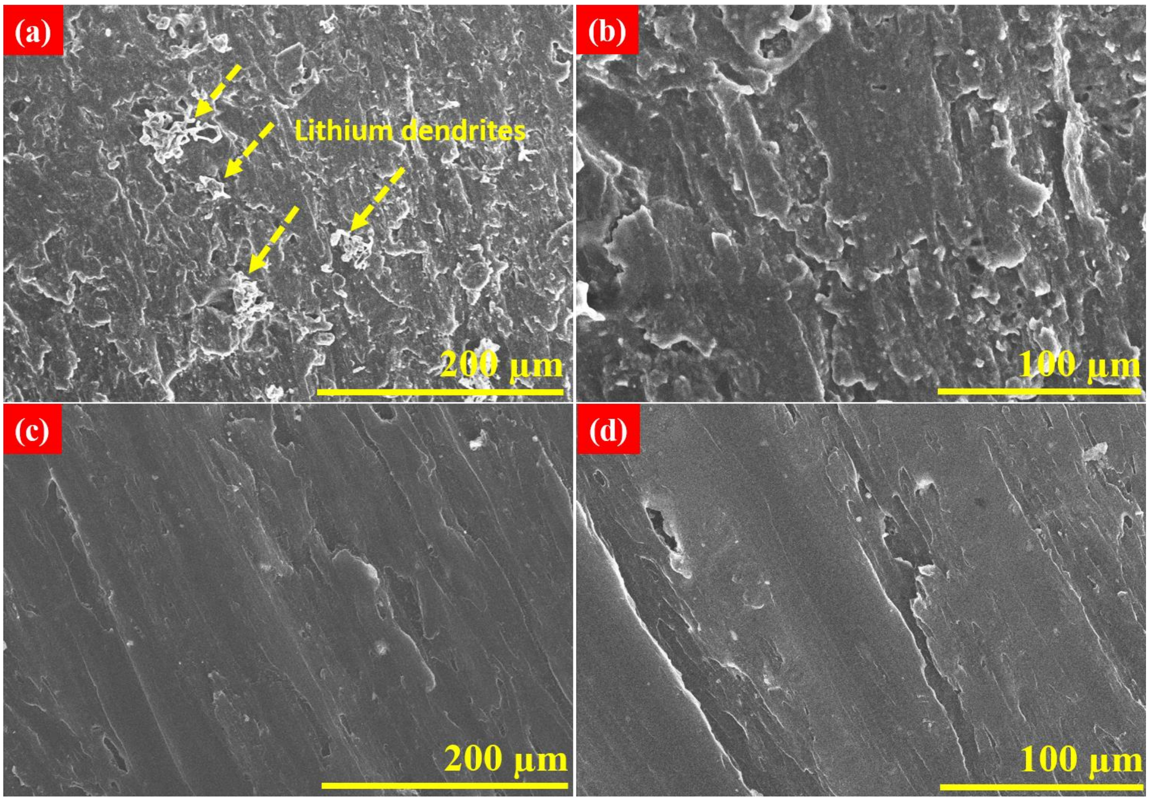

Fig.S9. SEM images of washed lithium surfaces after galvanostatic cycling in (a,b) PP separator and (c,d) Nb-PP separator containing lithium symmetric cells.

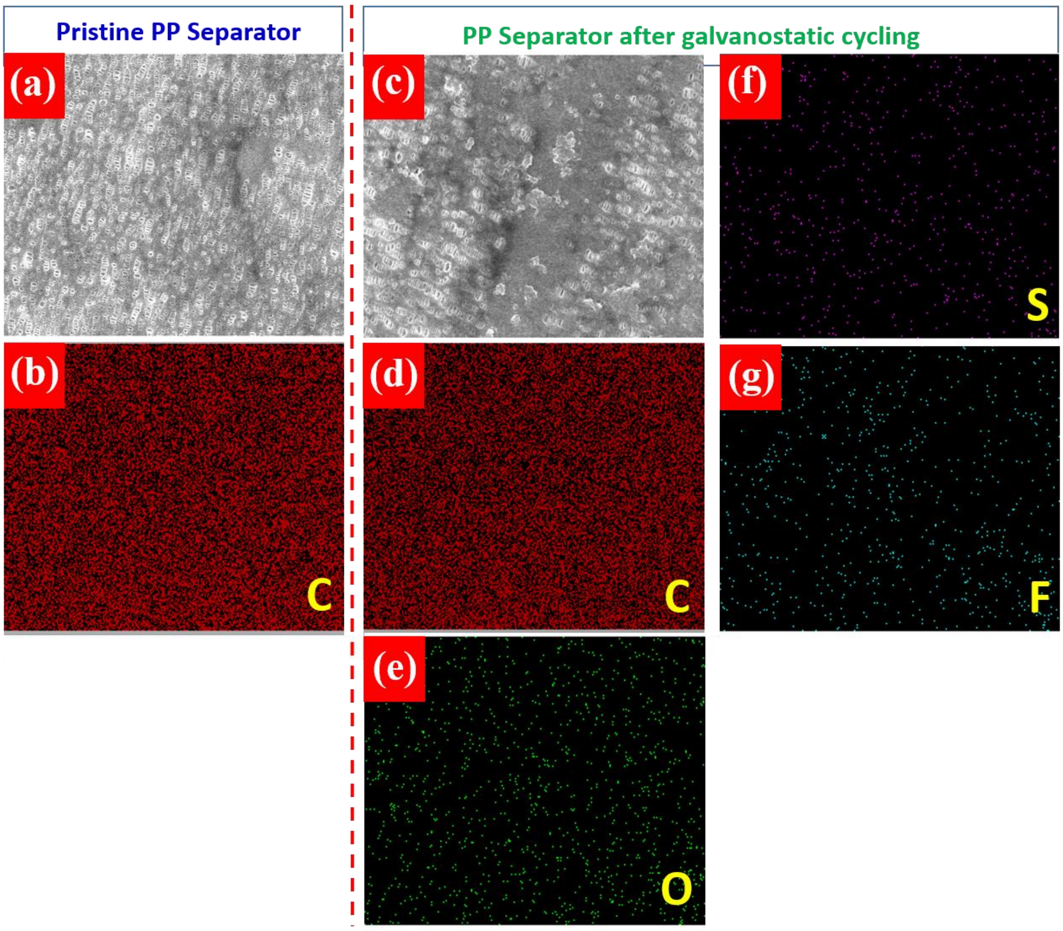

Fig. S10. EDS image of (a) pristine PP separator and (b) its corresponding elemental maps. EDS image of (c) PP separator after cycling and (d-g) its corresponding elemental maps.

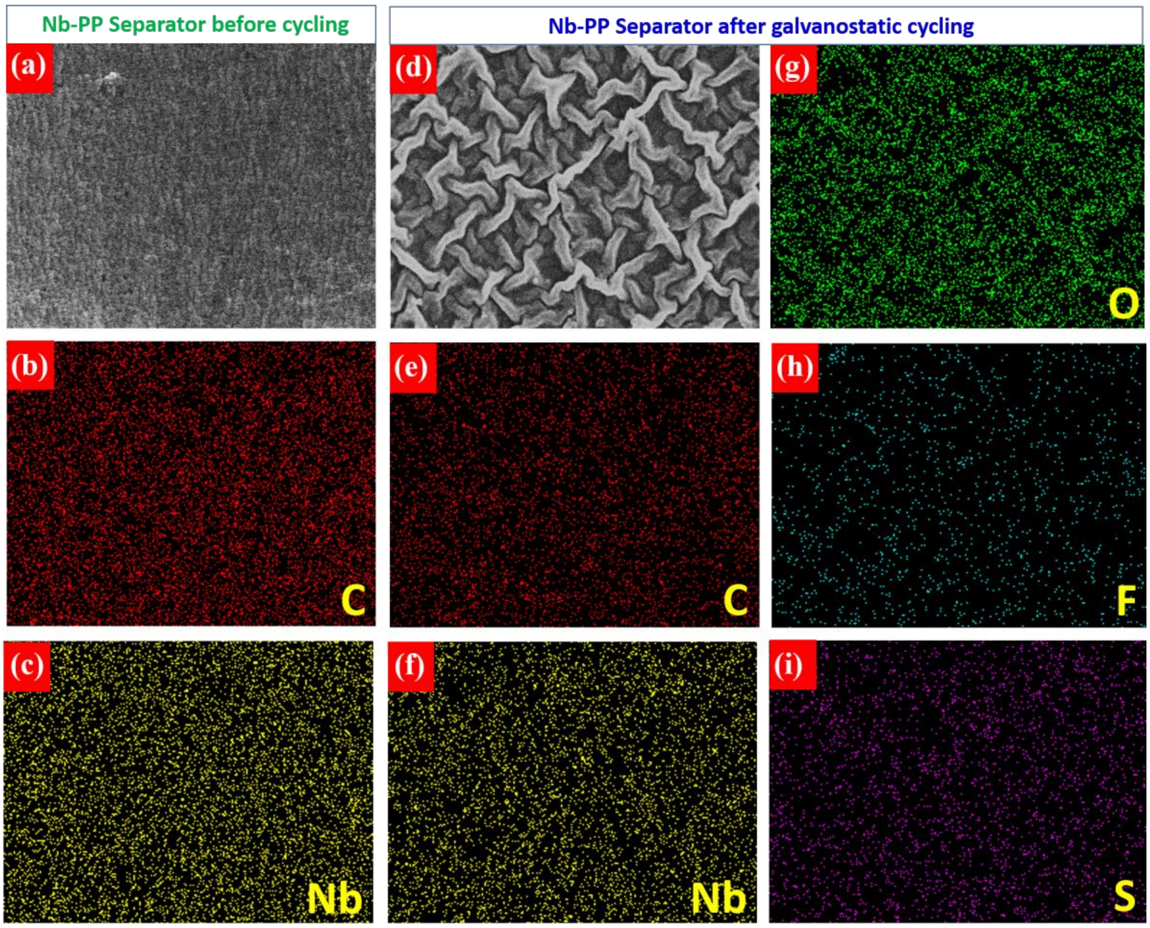

Fig. S11. EDS image of (a) Nb-PP separator and (b, c) its corresponding elemental maps. EDS image of (d) Nb-PP separator after cycling and (e-i) its corresponding elemental maps.

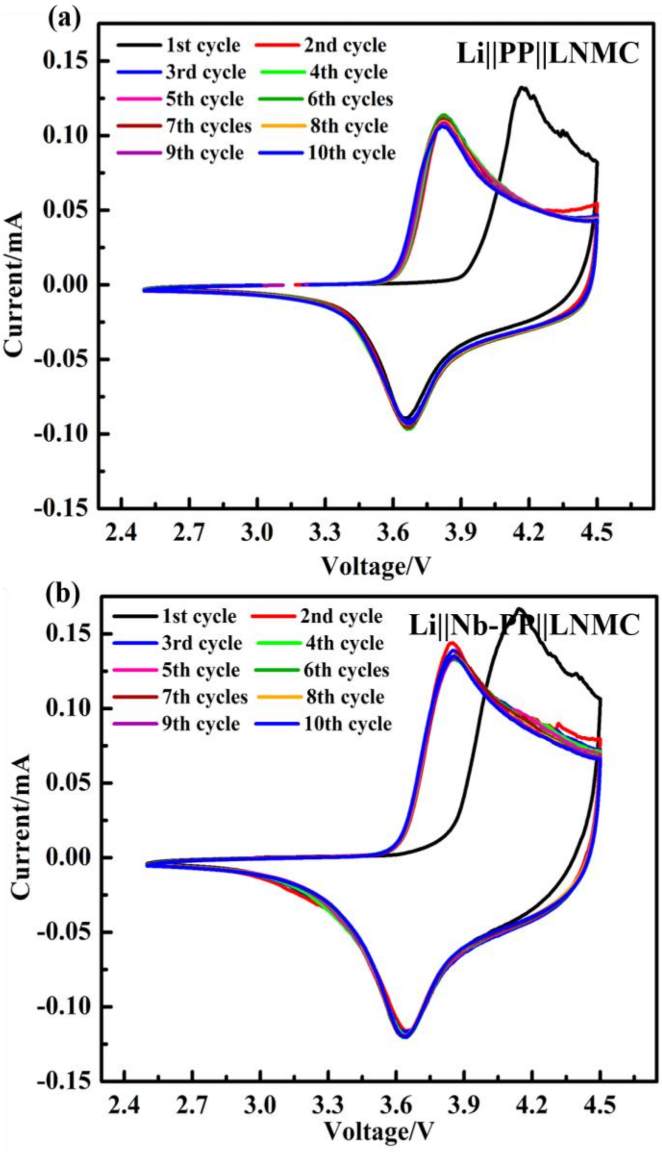

Fig. S12. CV measurements of PP and Nb-PP separator based half-cells containing LNMC cathode at 0.1 mVs^{-1} in 2.5 V to 4.5 V potential range. (a,b) 1st to 10th CV scans of respective cells.

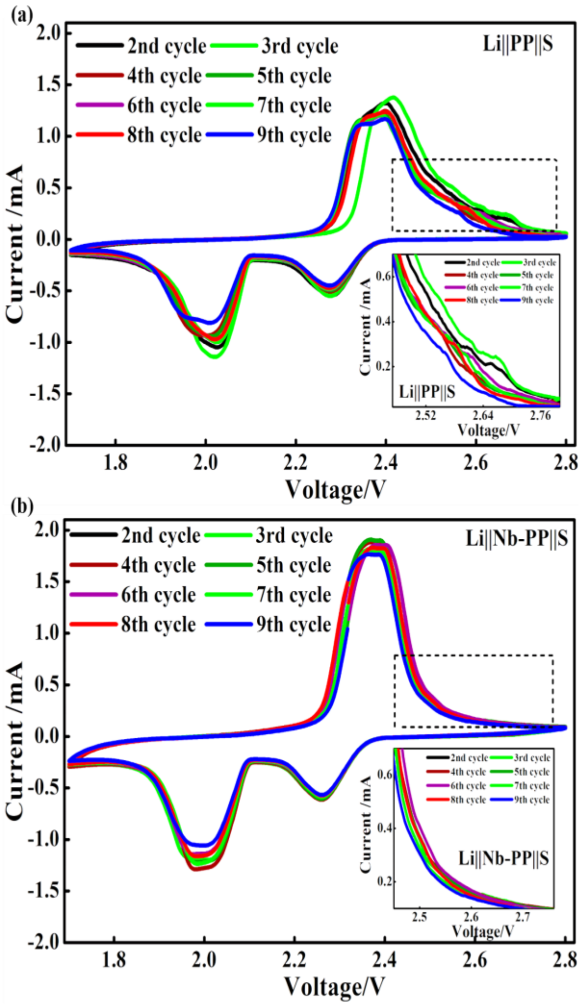

Fig. S13. CV measurements of (a) PP and (b) Nb-PP separator based half-cells at 0.1 mVs^{-1} in a potential window of 1.7 V to 2.8 V containing composite sulfur cathode. Inset in (a) represents the CV curves in anodic region showing small humps in voltage range 2.5 V to 2.7 V associated with polysulfide diffusion. Inset in (b) represents the CV curves in anodic region with no visible extra peaks in the higher voltage region.

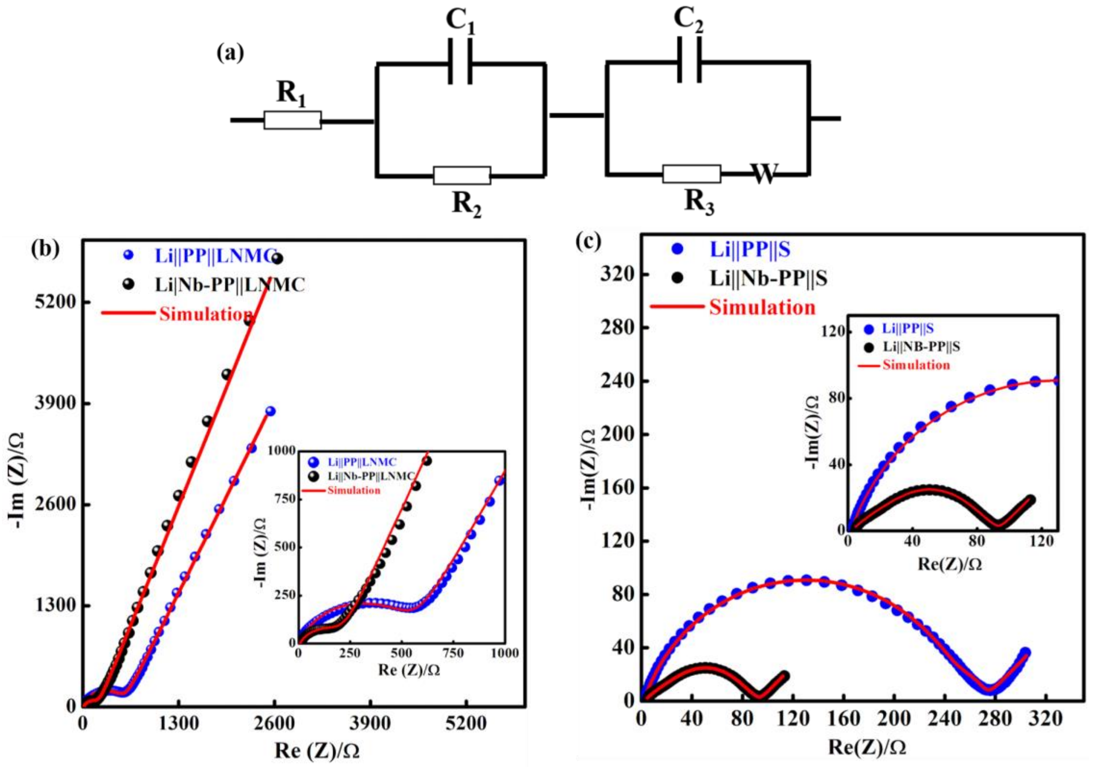

Fig. S14. (a) Circuit diagram for simulation of EIS data. (a) EIS plots of as-assembled Li||PP||LNMC and Li||Nb-PP||LNMC cells in 2 MHz to 0.01 Hz frequency range and 10 mV ac amplitude. (b) EIS plots of as-assembled Li||PP||S and Li||Nb-PP||S cells in 2 MHz to 0.01 Hz frequency range and 10 mV ac amplitude.
